# Conserved chloroplast genome sequences of the genus *Clerodendrum* Linn. (Lamiaceae) as a super-barcode

**DOI:** 10.1371/journal.pone.0277809

**Published:** 2023-02-09

**Authors:** Haimei Chen, Haodong Chen, Bin Wang, Chang Liu

**Affiliations:** 1 Institute of Medicinal Plant Development, Chinese Academy of Medical Sciences, Peking Union Medical College, Beijing, P. R. China; 2 School of Pharmacy, Xiangnan University, Chenzhou, Hunan, China; China National Rice Research Institute, CHINA

## Abstract

**Background:**

The plants of the genus *Clerodendrum* L. have great potential for development as an ornamental and important herbal resource. There is no significant morphological difference among many species of the genus *Clerodendrum*, which will lead to confusion among the herbs of this genus and ultimately affect the quality of the herbs. The chloroplast genome will contribute to the development of new markers used for the identification and classification of species.

**Methods and results:**

Here, we obtained the complete chloroplast genome sequences of *Clerodendrum chinense* (Osbeck) Mabberley and *Clerodendrum thomsoniae* Balf.f. using the next generation DNA sequencing technology. The chloroplast genomes of the two species all encode a total of 112 unique genes, including 80 protein-coding, 28 tRNA, and four rRNA genes. A total of 44–42 simple sequence repeats, 19–16 tandem repeats and 44–44 scattered repetitive sequences were identified. Phylogenetic analyses showed that the nine *Clerodendrum* species were classified into two clades and together formed a monophyletic group. Selective pressure analyses of 77 protein-coding genes showed that there was no gene under positive selection in the *Clerodendrum* branch. Analyses of sequence divergence found two intergenic regions: *trnH-GUG-psbA*, *nhdD*-*psaC*, exhibiting a high degree of variations. Meanwhile, there was no hypervariable region identified in protein coding genes. However, the sequence identities of these two intergenic spacers (IGSs) are greater than 99% among some species, which will result in the two IGSs not being used to distinguish *Clerodendrum* species. Analysis of the structure at the LSC (Large single copy) /IR (Inverted repeat) and SSC (Small single copy)/IR boundary regions showed dynamic changes. The above results showed that the complete chloroplast genomes can be used as a super-barcode to identify these *Clerodendrum* species. The study lay the foundation for the understanding of the evolutionary process of the genus *Clerodendrum*.

## Introduction

The plants of the genus *Clerodendrum* L. in the family Lamiaceae are about 400 species, mainly distributed in tropical and subtropical regions, with a few in temperate regions [1]. There are 34 species and 6 varieties are native to China, mostly in southwestern and southern China [2]. In China, most of the plants of the genus *Clerodendrum* are medicinal plants with the effects of clearing heat and detoxifying, dispelling wind and dampness, activating blood circulation and dispersing blood stasis, lowering blood pressure, anti-inflammatory and analgesic, which are commonly used to treat cold and high fever, rheumatic arthritis, bruises and injuries, hypertension, carbuncles and furuncles [3]. In Vietnam, *Clerodendrum cyrtophyllum* Turcz is used for the treatment of migraine, hypertension, pharyngitis, and rheumatic arthritis. Total phenols and flavonoids isolated from leaves of *C*. *cyrtophyllum* displayed potential antioxidant activity and anti-inflammatory activity [4]. In addition, many species of this genus have peculiar flower patterns, which are native to China, and have been domesticated and cultivated as ornamental plants including *Clerodendrum canescens* Wall., *Clerodendrum chinense* (Osbeck) Mabb, *Clerodendrum wallichii* Merr., *Clerodendrum trichotomum* Thunb., *Clerodendrum bungei* Steud., etc. Some *Clerodendrum* species include *Clerodendrum thomsoniae* Balf. f., *Clerodendrum speciosum* W. Bull, *Clerodendrum* quadriloculare (Blanco) Merr. and *Clerodendrum* splendens G. Don have been introduced from abroad as the ornamental plant. This shows that this genus has great potential for development as an important ornamental herbal medicine resource.

*Clerodendrum chinense* (Osbeck) Mabberley originating in Asia and commonly known as Chinese glory flower, is an evergreen shrub species in the Lamiaceae family [5]. It grows stout branches, usually up to about 2 meters tall. It produces fragrant, white-pink, rose-like, clusters of flowers followed by berries [5]. The roots and leaves of *C*. *chinense* are used as medicine to dispel wind and dampness, invigorate blood and strengthen muscles and bones [6]. The methanol extract of the leaves of *C*. *chinense* showed significant anti-inflammatory, analgesic and antipyretic effects [7]. The Centre for Agriculture and Bioscience International (CABI) classified *C*. *chinense* as a highly invasive species in tropical and subtropical ecosystems due to its aggressive suckering habit. It is also listed as an invasive species in Cuba, Puerto Rico, and the US Virgin Islands (https://candide.com/US/plants/). *C*. *chinense* is also classed as a major weed in Hawaii, Fiji, Western Samoa, and American Samoa and as a widespread exotic plant in the Lesser Antilles.

*Clerodendrum thomsoniae* Balf.f. native to West tropical Africa is a weak-stemmed, evergreen shrub with a more or less climbing habit. As a shrub, it usually reaches heights of over 1–2 metres. And when the twining habit is adopted, its stems can grow up to 7 metres [8]. The mashed leaves and flowers were used to treatment of bruises, cuts, skin rashes and sores. *C*. *thomsoniae* is widely cultivated as an ornamental and has been introduced in many places including America, the Galapagos Islands, and Australia.

Currently the utilization of traditional Chinese medicine resources generally has the problem of confusing products and pseudo, which will affect the quality of medicinal herbs. There is no significant morphological difference among many species of the genus *Clerodendrum*, that it is impossible to distinguish them in morphology. With the development of sequencing technology, complete chloroplast genome sequences have been used for the identification of medicinal plants, species classification and selection of superior germplasm. At present, although there are seven plastid chloroplast genomes of *Clerodendrum* species that have been released in Genbank [9–13], studies using complete plastid genomes for systematic analysis of the genus *Clerodendrum* are absent. In the present study, we reported the two complete plastome sequences of *C*. *chinense* and *C*. *thomsoniae* collected from Guangxi, China. The phylogenetic relationships of *Clerodendrum* species and its related genera were studied based on complete chloroplast genome sequence data. Systematically comparative analyses of the plastomes from *C*. *chinense*, *C*. *thomsoniae* and other *Clerodendrum* species were performed. The results will provide valuable information for future phylogenetic and taxonomic studies on the *Clerodendrum* species.

## Materials and methods

### Plant materials, DNA extraction, and sequencing

Young leaves of *C*. *chinense* and *C*. *thomsoniae* were freshly collected from the Guangxi Medical Botanical Garden, Nanning, China. Total DNA was extracted using the DNA extraction kit (Tiangen Biotech, Beijing, China) and stored at the Institute of Medicinal Plant Development (IMPLAD), Beijing, China with the ID number: Implad201910240 and Implad201910292, respectively. *C*. *chinense* and *C*. *thomsoniae* are not endangered or protected species. A total of 1μg DNA was used for DNA library construction, and the library with insert sizes of 500 bases was sequenced with Hiseq 2500 platform (Illumina, San Diego, CA, USA). A total of 16,943,658 and 17,455,640 paired-end reads were obtained with 150 bases long for *C*. *chinense* and *C*. *thomsoniae*.

### Genome assembly, annotation and repeat sequence analysis

The chloroplast genome was assembled from the whole genome sequencing data using NOVOPlasty (v.2.7.2) [14] with the seed sequence of *rbcL* gene from *Arabidopsis thaliana*. The annotation of the chloroplast genome was originally performed using CPGAVAS2 [15] and then curated using Apollo [16]. The genome sequence and annotations have been deposited in GenBank with accession numbers OM912811 and OM912812, respectively.

Three types of repeat sequences were identified. Perl script MISA (http://pgrc.ipk-gatersleben.de/misa/) [17] was used to identify simple sequence repeats (SSRs) with the parameters listed as follows: 10 repeat units for mononucleotide SSRs, 6 and 5 repeat units for di- and tri-nucleotide repeat SSRs, and 5 repeat units for tetra-, penta-, and hexanucleotide repeat SSRs. Tandem Repeats Finder was used with parameters of 2 for matches and 7 for mismatches and indels [18]. For the minimum alignment score and the maximum period, the size was set to 50 and 500, respectively. Palindrome and forward repeats were identified by the REPuter web service [19]. The minimum repeat size and the similarity cut-off were set to 20 bp and 90%, respectively.

### Phylogenetic analysis

To determine the phylogenetic position of *Clerodendrum* in the Lamiaceae, 46 species with released complete chloroplast genome were selected. Among the 46 species, 23 species were in the subfamily Ajugoideae, which contained four plant tribes including Ajugeae (7 species), Clerodendreae (9 species), Teucrieae (5 species) and Rotheceae (2 species). *Mazus pumilus* and *Phryma leptostachya* were set as outgroups. Firstly, the released complete chloroplast genomes were restarted with the *trnH* gene using SeqKit v0.12.1 [20]. Secondly, the multiple sequence alignments of the complete sequences of chloroplast genomes were performed derived from 46 species using MAFFT v6.861b [21] with default parameters. Then the conserved blocks of multiple sequence alignment were selected using Gblocks v0.91b [22] with default parameters. The dataset of conserved blocks was subjected to phylogenetic analysis using IQ-TREE v1.6.12 [23] with the TVM+F+R3 model and ultrafast bootstrap 1000 replicates. The tree file with nwk format was visualized by MEGA-X [24]. The taxon information of species used for phylogenetic analysis was listed in S1 Table.

### Selective pressure analysis

As synonymous substitutions accumulate nearly neutrally, non-synonymous substitutions are subject to selective pressures of varying degrees and directions (positive or negative). In general, the ratio of nonsynonymous to synonymous substitution (ω) measures the levels of selective pressure operating in a protein coding gene. In the evolutionary analysis above, we found that the chloroplast whole genome sequences of *Clerodendrum lindleyi* (NC_056199.1) and *Clerodendrum bungei* (NC_056141.1) differed by only 4 bases, so we excluded the sequence of *C*. *bungei* for the further analysis. To test which genes were subject to positive selection at the genus *Clerodendrum* branch, we conducted the selection analysis of protein-encoding genes derived from Ajugoideae branch. A total of 77 protein-encoding genes among 21 species belonging to the subfamily Ajugoideae were extracted and subjected to positive selection using our custom script. In brief, the selective pressure analysis was conducted using HyPhy v2.3.1120180415beta (MP) with the adaptive branch-site random effects likelihood (aBSREL) model [25] (http://www.hyphy.org/). Significance was assessed using the Likelihood Ratio Test at a threshold of p ≤ 0.05, after correcting for multiple testing. Finally, the sites that were subject to positive selection along Ajugoideae branch were viewed via this website http://vision.hyphy.org/.

### Hypervariable region analysis

The pairwise distance was determined using the Distmat program that was implemented in EMBOSS (v6.3.1) [26] with the Kimura 2-parameters (K2p) evolution model [27] for intergenic regions of eight *Clerodendrum* species. To determine the threshold for the K2p distance to be a hypervariable region, we calculated the average and the standard deviation for all the K2p values. The average + 2*STD was then set as the threshold.

### Boundary analysis of *Clerodendrum* plastomes

To obtain the gene distribution on large single copy region (LSC), small single copy region (SSC), inverted repeat a (IRa), and IRb borders, the location of genes on the boundaries was visualized using IRSCOPE [28]. The GenBank files of eight *Clerodendrum* chloroplast genome were used for the input file of IRSCOPE. The genomes were restarted with the *trnH* gene and re-annotated using CPGAVAS2 with custom reference.

## Results

### Chloroplast genome

**Fig 1:**
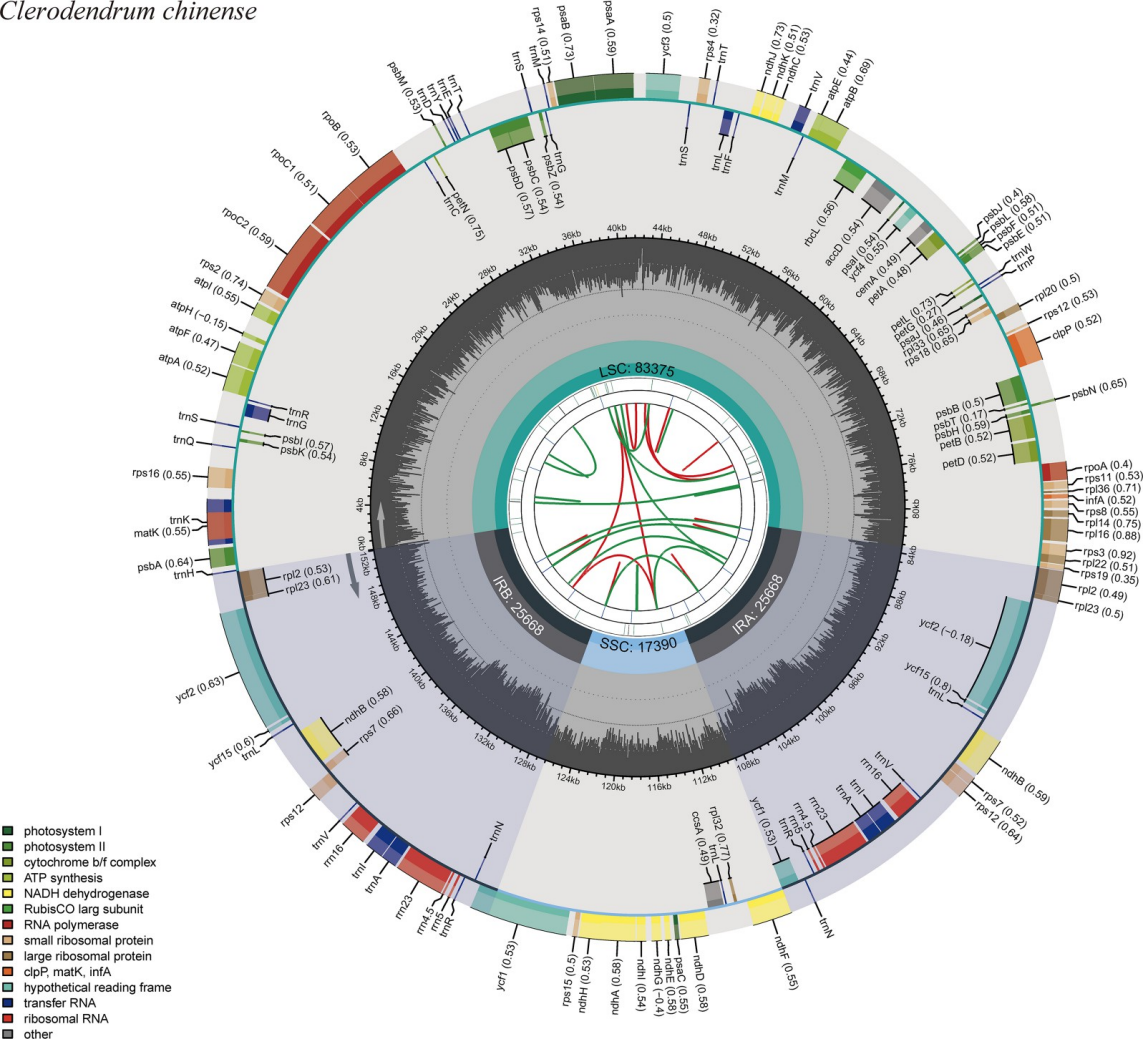
Gene map of the *C*. *chinense* plastome. There are six rings on the diagram from the center outwards. The first ring is used to indicate the position of forward (red arcs) and reverse (green arcs) repeats. The second ring is used to indicate tandem repeats (short columns). The third ring is used to indicate microsatellite repeats (short columns). The fourth shaded ring indicates each part and the length of SSC, IRa, IRb, and LSC regions. The optional shaded area stretching from the inner sphere toward the outer circle marks the IR regions The fifth ring indicates the GC content of the chloroplast genome. The outer ring shows the gene names and their optional codon usage bias. The genes are colored based on their functional categories.

Chloroplast DNA of *C*. *chinense* and *C*. *thomsoniae* as circular molecules, and the total length of its genome sequence is 152,101 bp and 151,053 bp. They all have a conserved tetrad structure consisting of a LSC region, a SSC region and a pair of IR regions. The length of LSC, SSC and IR regions of *C*. *chinense* were 83,375 bp, 17,390 bp and 25,668bp respectively (Fig 1). And the length of LSC, SSC and IR regions of *C*. *thomsoniae* were 82,742 bp, 17,225 bp and 25,543 bp respectively (S1 Fig). The overall G/C content of the chloroplast genome of *C*. *chinense* and *C*. *thomsoniae* was 38.15% and 38.21%. The G/C content of the IR region of *C*. *chinense* is 43.29% which is higher than that of the SSC region (31.93%) and the LSC region (36.28%). The G/C content of the IR region of *C*. *thomsoniae* is 43.33% which is also higher than that of the SSC region (32.08%) and the LSC region (36.32%).

### Coding genes

The chloroplast genomes of *C*. *chinense* and *C*. *thomsoniae* all encode a total of 131 genes, including 112 unique genes, including 83 protein-coding genes (80 unique genes), 35 genes encoding transfer RNA (28 unique tRNA genes) and 8 genes encoding ribosomal RNA (4 unique rRNA genes) (S2 Table). Eight of the protein-coding genes (*ndhB*, *rpl*2, *rpl*23, *rps*12, *ycf*2, *rps*7, *ycf15*, *ycf1*), seven tRNA-coding genes (*trnA-UGC*, *trnE-UUC*, *trnL-CAA*, *trnM-CAU*, *trnN-GUU*, *trnR-ACG*, *trnV-GAC*), and four rRNA-coding genes (*rrn4*.*5*, *rrn5*, *rrn16* and *rrn23*) were in the IR region. Nine protein-coding genes (*rps16*, *atpF*, *rpoC1*, *rpl16*, *petB*, *petD*, *rpl2*, *ndhB*, *ndhA*) contain one intron, and two protein-coding genes (*ycf*3, *clpP*) containing 2 introns, and 6 tRNA-encoding genes (*trnK-UUU*, *trnS-CGA*, *trnL-UAA*, *trnV-UAC*, *trnE-UUC*, *trnA-UGC)* containing 1 intron (S3 and S4 Tables). The length of the coding sequence (CDS) in the chloroplast genome of *C*. *chinense* was 80,663 bp, accounting for 53.03% of the total genome length. rRNA genes were 9,050 bp, accounting for 5.95% of the total genome length, while the length of tRNA genes was 2,642 bp, accounting for 1.74% of the total genome length. The non-coding regions of the chloroplast genome of *C*. *chinense* mainly consisted of introns and spacer regions, which accounted for 39.28% of the entire genome length. The length of CDS in the chloroplast genome of *C*. *thomsoniae* was 80,117 bp, accounting for 53.04% of the total genome length. rRNA genes were 9,050 bp in length, accounting for 5.99% of the total genome, while the length of tRNA genes was 2,642 bp, accounting for 1.75% of the total genome length. The non-coding regions of the chloroplast genome of *C*. *thomsoniae* mainly consisted of introns and spacer regions, which accounted for 39.22% of the entire genome length.

### Repeat sequence

In this study, three types of repeat sequence were identified namely microsatellite sequence repeat, tandem repeat and dispersed repeat, which can be used as a molecular marker to distinguish species.

In the *C*. *chinense* chloroplast genome, the type of microsatellite repeats was dominated by A/T with 36 times, followed by AT/AT with 3 times, C/G with 4 times, and AAT/ATT with 1 time (S5 Table). In the *C*. *thomsoniae* chloroplast genome, the type of microsatellite repeat sequence was dominated by A/T with 40 times, followed by AT/AT times, and AAT/ATT with 1 time (S6 Table). In addition, the distributions of the identified SSRs were also investigated. In the present study, SSRs were less abundant in the CDS than in the intergenic spacer (IGS) of the chloroplast genome of *C*. *chinense* and *C*. *thomsoniae* (S7 and S8 Tables).

A tandem repeat sequence is a sequence consisting of multiple consecutive arrangements of repeat units on a chromosome. 13 tandem repeats were found in the *C*. *chinense* chloroplast genome (S9 Table) and 8 tandem repeats were found in the *C*. *thomsoniae* (S10 Table) chloroplast genome, meeting two criteria of total length over 20bp and ≥90% similarity between repeat units.

Dispersed repeats are a different type of repetitive sequence from tandem repeats in that the repeat units are distributed throughout the genome. There are four types of dispersed repeats, which are forward, reverse, complement and palindromic repeats. The *C*. *chinense* chloroplast genome has 18 palindromic repeats and 14 forward repeats with a threshold E-value of less than 1E-4 (S11 Table). The *C*. *thomsoniae* chloroplast genome has 16 palindromic repeats and 11 forward repeats (S12 Table).

These repeats identified in this study will provide valuable resources for species identification and population studies of *Clerodendrum* spp.

### Phylogenetic tree

**Fig 2:**
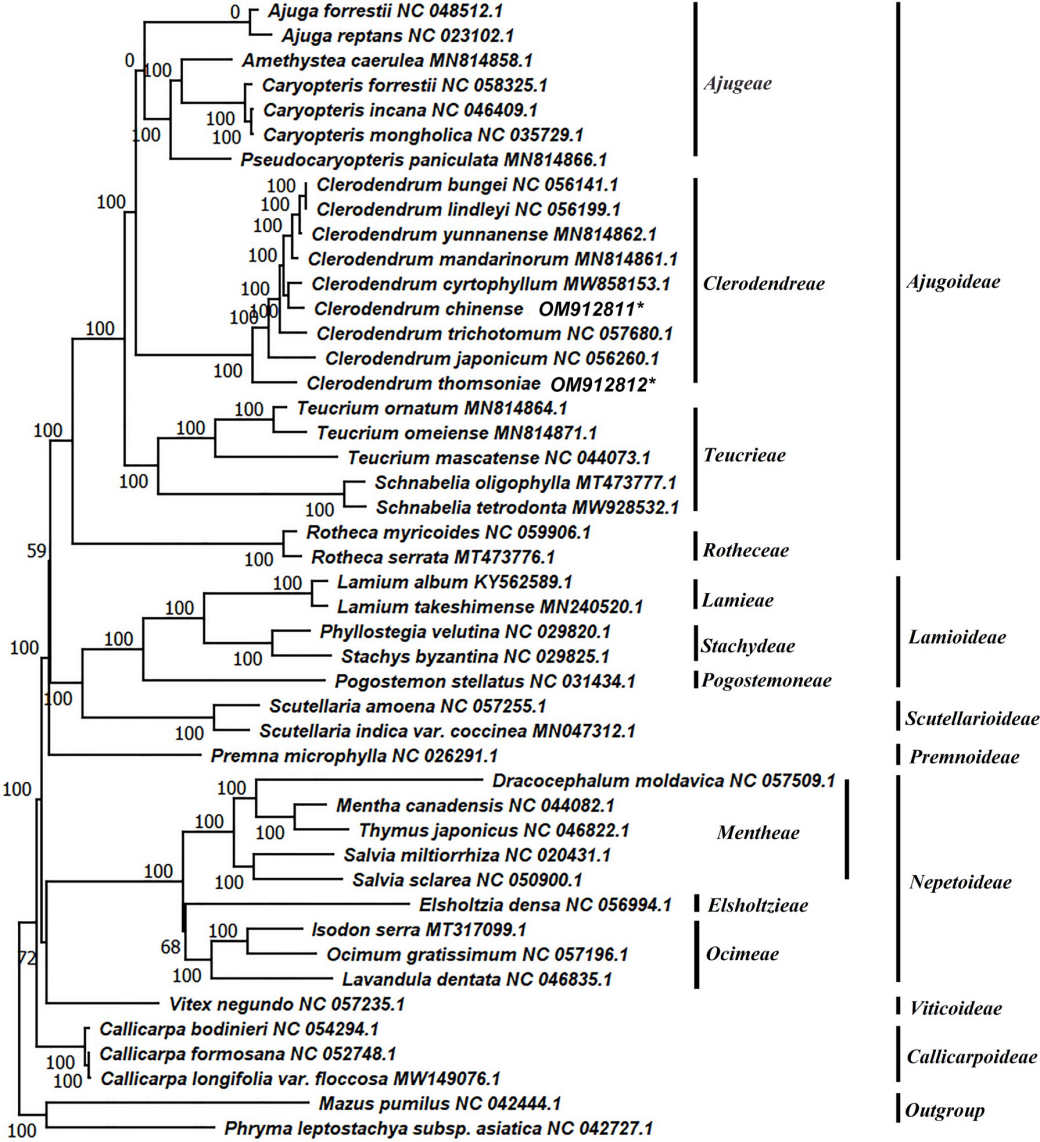
Evolutionary tree of family Lamiaceae. The phylogenetic results included 44 species within family and two out-group species. *Mazus pumilus* and *Phryma leptostachya* were set as the outgroups. The species marked with an asterisk are *C*. *Chinense* and *C*. *thomsoniae*, whose chloroplast genomes were firstly reported. The right of the panel is the name of tribe and subfamily according to the species. Numbers above the branches are the bootstrap support values.

To identify the phylogenetic positions of the nine *Clerodendrum* spp. within the Liliaceae, we obtained 44 complete chloroplast genome sequences belonging to 7 subfamilies of the family Lamiaceae including Ajugoideae, Lamiodeae, Scutellarioideae, Premnoideae, Viticoideae, Callicarpoideae from the NCBI database (S1 Table). In addition, 23 species belonging to four tribes of Ajugoideae were selected, namely Ajugeae, Clerodendreae, Teucrieae, Rotheceae. *Mazus pumilus* and *Phryma leptostachya* were set as outgroup. Multiple sequence alignment of the complete genome was carried out by MAFFT. The best-fit model TVM+F+R3 was obtained by IQ-tree screening, and the phylogenetic tree was constructed by the maximum Likelihood method. All the species from *Clerodendrum* were clustered together and formed a monophyletic group. *C*. *chinense and C*. *cyrtophyllum* were clustered together to be the sister of *C*. *bungei*, *C*. *lindleyi*, *C*. *yunnanense* and *C*. *mandarinorum*. *C*. *thomsoniae* was at the basal clade of *Clerodendrum* (Fig 2). The distribution range of *C*. *chinense*, *C*. *cyrtophyllum*, *C*. *bungei*, *C*. *lindleyi*, *C*. *yunnanense*, *C*. *mandarinorum*, *C*. *trichotomum* and *C*. *japonicum* was east of Asia. While the distribution range of *C*. *thomsoniae* was west tropical Africa. Our results showed that the Asian clade and African clade are sister groups and together form a monophyletic group. These results will provide an important reference and foundation for species identification and phylogeny of *Clerodendrum* plants.

### Selective pressure analysis

Selective pressure analyses of 77 protein-coding genes in the subfamily Ajugoideae plastomes showed that six genes (*clpP*, *ndhA*, *ndhF*, *rpoC2*, *rpl22* and *ycf1*) were found to have evolved under positive selection in Ajugoideae branch. And the significance and number of rate categories inferred at the Ajugoideae branch are provided in Table 1. The longest optimized branch length of the Ajugoideae branch is 0.0113 for the *ycf1* gene. And there was no gene under positive selection in the *Clerodendrum* branch.

**Table 1:** The results of positive selection genes at *Clerodendrum* branch.

Species	Gene	B	LRT	Test p-Value	Uncorrected p-Value	ω Distribution over Sites
NC_048512.1	*clpP*	0.0024	19.6771	0.0007	0.0000	ω_1_ = 0.00(93%) ω_2_ = 3630(6.9%)
MW928532.1	*ndhA*	0.0037	21.7134	0.0003	0.0000	ω_1_ = 0.00(98%) ω_2_ = 10000(2.4%)
NC_035729.1	*ndhF*	0.0005	35.8844	0.0000	0.0000	ω_1_ = 0.00(99%) ω_2_ = 2410(0.85%)
MT473777.1	*rpoC2*	0.0032	34.9383	0.0000	0.0000	ω_1_ = 0.235(100%) ω_2_ = 10000(0.42%)
NC_046409.1	*rpoC2*	0.0004	19.8517	0.0006	0.0000	ω_1_ = 0.0838(99%) ω_2_ = 1970(0.54%)
MN814858.1	*rpl22*	0.0099	11.4022	0.0000	0.0000	ω_1_ = 0.00(87%) ω_2_ = 24.1(13%)
MN814866.1	*ycf1*	0.0113	26.2515	0.0000	0.0000	ω_1_ = 0.0621(100%) ω_2_ = 10000(0.40%)

B: Optimized branch length; LRT: Likelihood ratio test statistic for selection; Test *p*-value: *p*-value corrected for multiple testing; Uncorrected *p*-value: Raw *p*-value without correction for multiple testing.

### Hypervariable regions

**Fig 3:**
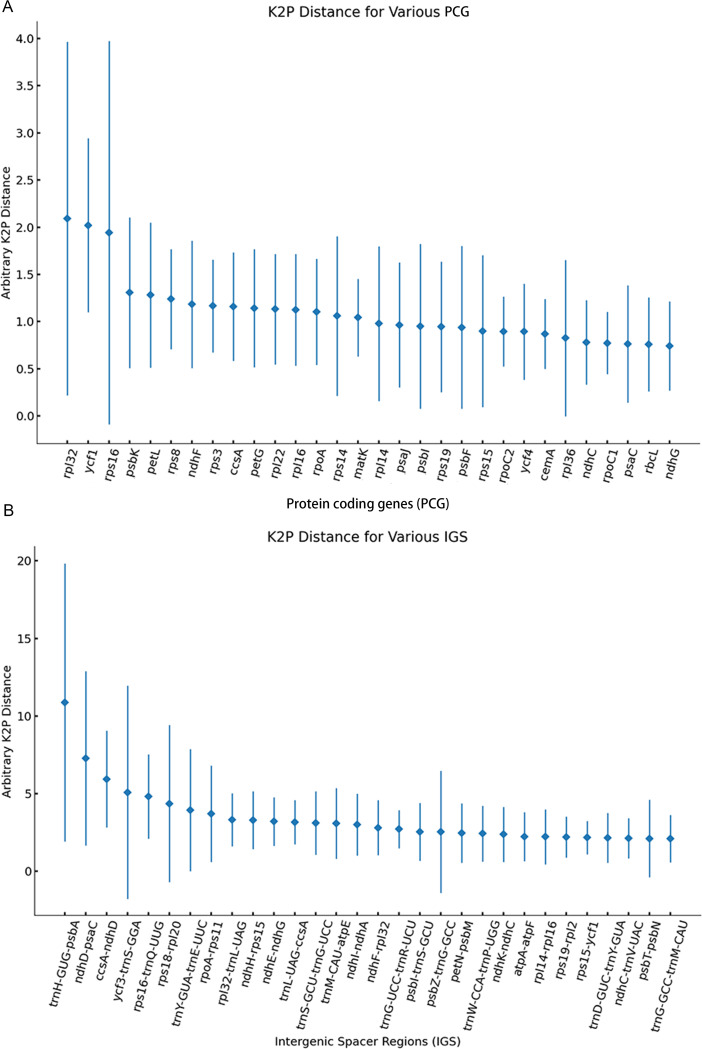
Results of genetic distance analysis of protein coding genes (A) and intergenic regions (B) in *Clerodendrum* species. Comparison of the variability of PCG and IGS regions among *C*. *chinense*, *C*. *cyrtophyllum*, *C*. *lindleyi*, *C*. *yunnanense*, *C*. *mandarinorum*, *C*. *trichotomum*, *C*. *thomsoniae and C*. *japonicum*. The X-axis indicates the name of PCG and IGS regions. The top thirty PCG or ICG of K2p distances were showed in panel A and panel B. And the Y-axis shows the range of K2p distances between different pairs of species. The diamond shows the average K2p distance, respectively.

The pairwise comparison of protein coding genes (PCGs) and intergenic spacer regions among the eight *Clerodendrum* species was conducted to identify hypervariable regions using the Kimura 2-parameter (K2p) model. The high variable region threshold of K2p for PCG and IGS are 2.13 and 6.39, respectively. The K2p distance ranged from 0.00 to 6.49 among 77 PCGs of the eight *Clerodendrum* species. Among them, the *rpl32*, *ycf1* showed the average distances of 2.09 and 2.02 among the eight *Clerodendrum* species, which were all lower than the K2p value threshold for PCG (Fig 3A). The K2p distance ranged from 0.00 to 33.21 among 117 IGSs of the eight *Clerodendrum* species. Among them, the IGS regions *trnH-GUG-psbA*, *nhdD*-*psaC* showed average distances of 11.97 and 7.22 among the eight *Clerodendrum* species, respectively (Fig 3B). However, *trnH-GUG-psbA* region could not be used to distinguish the *C*. *yunnanense* and *C*. *lindleyi* with 99% sequence identities. The sequence similarity of the *nhdD*-*psaC* region among the three species *C*. *trichotomum*, *C*. *mandarinorum* and *C*. *Chinense* is 100%, which making it impossible to distinguish between these species. In conclusion, there was no single region can be used as molecular markers for evaluating plant phylogeny at low taxonomic levels and for DNA barcoding in *Clerodendrum*.

### Dynamic changes in the IR region of *Clerodendrum* plastome

**Fig 4:**
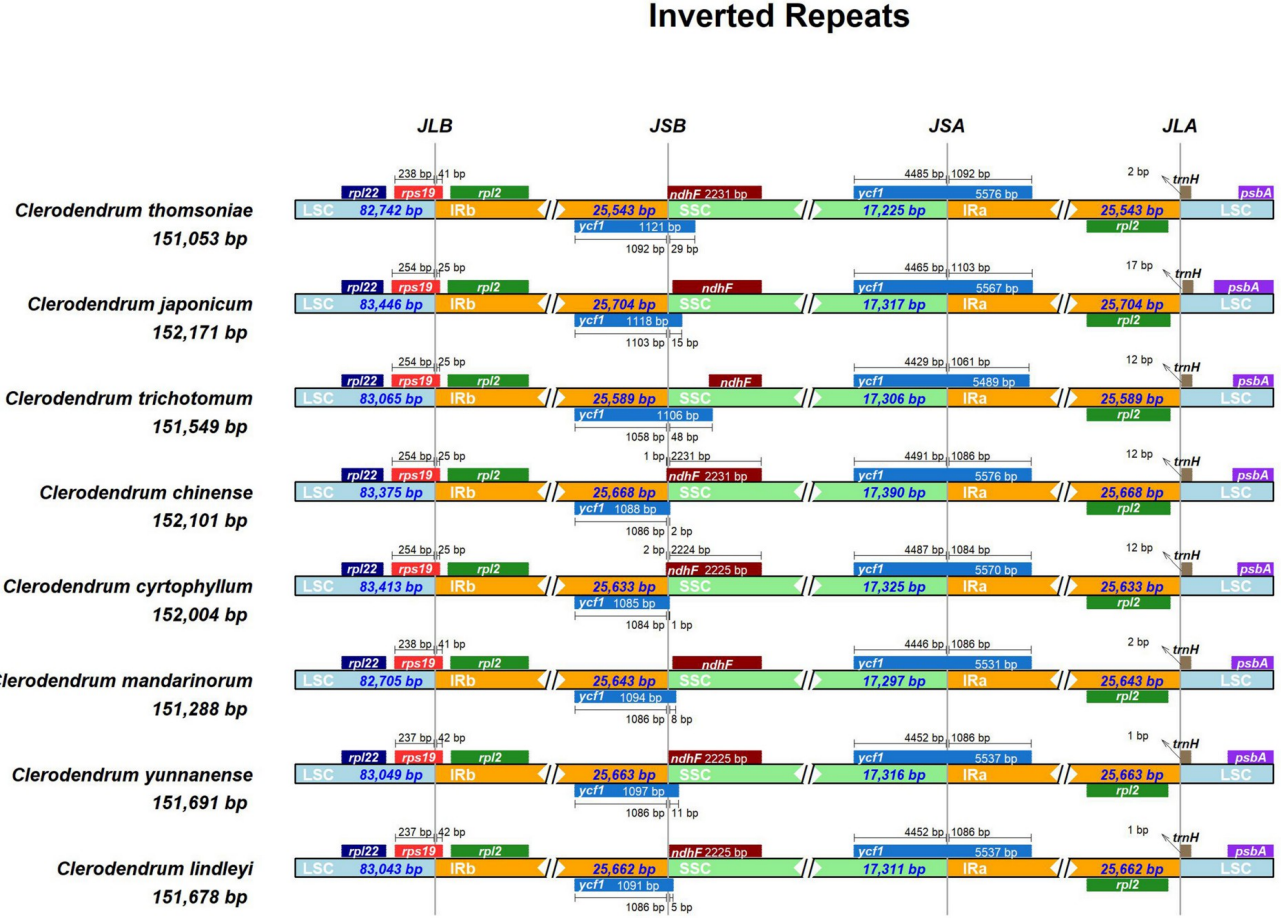
Boundary analysis of the single copy region and inverted repeated region among 8 *Clerodendrum* species. The name of each species and its corresponding length of chloroplast genome sequence are described on the left side of each track. Genes shown above the lines are transcribed forward while genes shown below the lines are transcribed reversely. Light blue, orange, and green box represent LSC, IR and SSC region, respectively. The numbers on the box indicate the length of each region. The number near the arrow shows the distance between the start or end coordinates of a given gene and the corresponding junction site. JLB (IRb/LSC), JSB (IRb/SSC), JSA (SSC/IRA) and JLA (IRa/LSC) represent the junction between corresponding regions in the genome respectively.

The expansion/contraction of the IR regions [29] is one reason for variation in the size of plastomes. Based on the distances between the border of *rps19* and the IR/LSC junction, the eight *Clerodendrum* species were classified into three groups (Fig 4). The *rps19* gene of the first group was 238bp and 41bp in the LSC and IR regions, respectively, which included *C*. *mandarinorum* and *C*. *thomsoniae*. The second group included *C*. *japonicum*, *C*. *trichotomum*, *C*. *Chinense*, *C*. *cyrtophyllum*, in which the *rps19* gene was 254bp and 25bp in the LSC and IR regions, respectively. The third group included *C*. *yunnanense* and *C*. *lindleyl*, in which the *rps19* gene was 237 bp and 42 bp in the LSC and IR regions, respectively. Another interesting observation is that the *ycf1* gene is localized in the IRb/SSC junction. The *ycf1* gene sequence is 1092 bp for *C*. *thomsoniae*, 1103 bp for *C*. *japonicum*, 1061 bp for C. *trichotomum*, 1084 bp for *C*. *cyrtophyllum* and 1086 bp for *C*. *Chinense*, *C*. *mandarinorum*, *C*. *yunnanense* and C. *lindleyl*. The above result showed that the expansion or contraction of IR boundaries in *Clerodendrum* species were dynamic events.

## Discussion

Here, we studied two *Clerodendrum* species, *C*. *chinense* and *C*. *thomsoniae*. We carried out a detailed analysis of the genome features, and performed the phylogenetic analysis with whole chloroplast genome sequences. Phylogenetic analyses showed that nine *Clerodendrum* species were on a monophyletic clade, with 100% bootstrap values. Analyses of selective pressure revealed that there was no gene undergoing positive selection in *Clerodendrum* branch. Analyses of sequence divergence found two sites: IGS (*trnH-GUG-psbA*) and IGS (*nhdD*-*psaC*) had a high degree of variations. Analysis of the plastomic structure at the LSC/IR and SSC/IR boundary regions showed dynamic events.

With the development of molecular systematic methods, the delimitation of *Clerodendrum* continues to be modified. Four large discrete clades (Clades I-IV) within *Clerodenidrum* s.l. were identified using chloroplast DNA (cpDNA) restriction site data [30] and showed that *Clerodendrum* s.l.is polyphyletic. Among them, clades I and II comprise Asian and African taxa respectively. Clade III comprises coastal. Clade IV, comprising subg. *Cyclonema* and sect. *Konocalyx* (subg. *Clerodendrum* pro parte) emerges as a lineage distinct from the rest of *Clerodendrum*. *Cyclonema* and *Konocalyx* form a clade distinct from *Clerodendrum* s.s., which has been identified as *Rotheca* Raf using the internal transcribed spacers (ITS) of the nuclear ribosomal DNA [31]. To clarify the positions of these four genera relative to *Clerodendrum*, ITS and chloroplast *ndhF* sequence data were used. The phylogenetic results showed that the Australian monotypic genus *Huxleya* evolved from within *Clerodendrum* and was sunk into *Clerodendrum* with a new combination, *Clerodendrum linifolium* [1]. Yuan et al. [32] proposed to separate the Pantropical Coastal clade and *C*. *spinosum* by reviving the genera *Volkameria* (including *Huxleya*) and *Ovieda*, and restricting *Clerodendrum* to the Asian and African clades based on the phylogenetic study of four relatively fast-evolving chloroplast DNA regions, *trnT-L*, *trnL-F*, *trnD-T*, and *trnS-fM*. In this paper, we examine the taxonomic positions of seven more labiate genera related to *Clerodendrum*: *Ajuga*, *Amethystea*, *Caryopteris*, *Teucrium*, *Pseudocaryopteris*, *Rotheca*, and *Schnabelia*. We also examine the positions of these taxa in the broader context of subfamily Ajugoideae. The results corroborate previous studies that the Asian clade and African clade are sister groups and together form a monophyletic group.

Gogoi et al. [33] evaluated four barcode candidates (*ITS2*, *matK*, *rbcL*, *ycf1*) and their combinations in different *Clerodendrum* species of Northeast India. The reliability of these barcodes to distinguish the species was evaluated by genetic pairwise distances, intra- and inter-specific diversity, barcode gap, and phylogenetic tree-based methods. The results showed that the combination of *ITS*2 + *matK* was suggested to be the core barcode for *Clerodendrum*. Three chloroplast sequences, *psbA*-trn*H*, *rbcL*, *matK*, two nuclear ribosomal DNA *ITS*2 and *ITS* have been used to be as DNA barcodes for identifying medicinal plant species in Verbenaceae [34], which proposes that the combination of *ITS2* and *psbA*-*trnH* sequence is promising for the identification of the species in Verbenaceae. In this study, we found that the K2p values were particularly high for the two IGS regions: *trnH-GUG*-*psbA*, *nhdD*-*psaC*. Meanwhile, there were no hypervariable regions identified for the PCG. However, the sequence identities of *trnH*-*GUG*-*psbA* and *nhdD*-*psaC* are greater than 99% among some species, which will result in the two *IGSs* not being used to distinguish *Clerodendrum* species.

## Conclusions

The complete plastomes of *C*. *thomsonia* and *C*. *Chinense* are reported for the first time in this study. The study showed that the complete chloroplast genomes can be used as a super-barcode to identify these *Clerodendrum* species. The results obtained from these studies will contribute to our understanding of *Clerodendrum* classification, plastome evolution, and the discrimination of medicinal products derived from *Clerodendrum* species.

## Supporting information

S1 Table The taxonomy of related species for phylogenetic analysis. (DOCX)

S2 Table List of genes in the chloroplast genome of *C*. *chinense* and C. *thomsoniae*. (DOCX)

S3 Table Intron and exon positions and lengths of chloroplast genes in *C*. *chinense*. (DOCX)

S4 Table Intron and exon positions and lengths of chloroplast genes in *C*. *thomsoniae*. (DOCX)

S5 Table Statistics on the number of microsatellites repeat sequences in the chloroplast genome of *C*. *chinense*. (DOCX)

S6 Table Distribution of nucleotide SSR sequences in the chloroplast genome of *C*. *chinense*. (DOCX)

S7 Table Statistics on the number of microsatellite repeat sequences in the chloroplast genome of *C*. *thomsoniae*. (DOCX)

S8 Table Distribution of nucleotide SSR sequences in the chloroplast genome of *C*. *thomsoniae*. (DOCX)

S9 Table Tandem repeat sequence statistics of the chloroplast genome of *C*. *chinense*. (DOCX)

S10 Table Tandem repeat sequence statistics of the chloroplast genome of *C*. *thomsoniae*. (DOCX)

S11 Table Characteristic values of scattered repetitive sequences in the chloroplast genome of *C*. *chinense*. (DOCX)

S12 Table Characteristic values of scattered repetitive sequences in the chloroplast genome of *C*. *thomsoniae*. (DOCX)

S1 Fig Map of the chloroplast genome of *C*. *thomsoniae* using CPGview-RSG. (DOCX)
