# Supplementary material for: Conserved chloroplast genome sequences of the genus Clerodendrum Linn. (Lamiaceae) as a super-barcode
Source: PLoS One. 2023 Feb 9;18(2):e0277809. doi: 10.1371/journal.pone.0277809 (PMC9910634; doi:10.1371/journal.pone.0277809)
Supplement: S1 Table — (DOCX) [file pone.0277809.s001.docx]

**S1 Table. The taxonomy of related species for phylogenetic analysis**

| Subfamily | Tribe | Genus | Species | Accession number |  |
| --- | --- | --- | --- | --- | --- |
| Ajugoideae | Ajugeae | *Ajuga* | *Ajuga forrestii* | NC_048512.1 |  |
| Ajugoideae | Ajugeae | *Ajuga* | *Ajuga reptans* | NC_023102.1 |  |
| Ajugoideae | Ajugeae | *Amethystea* | *Amethystea caerulea* | MN814858.1 |  |
| Callicarpoideae | NA | *Callicarpa* | *Callicarpa bodinieri* | NC_054294.1 |  |
| Callicarpoideae | NA | *Callicarpa* | *Callicarpa formosana* | NC_052748.1 |  |
| Callicarpoideae | NA | *Callicarpa* | *Callicarpa longifolia* | MW149076.1 |  |
| Ajugoideae | Ajugeae | *Caryopteris* | *Caryopteris forrestii* | NC_058325.1 |  |
| Ajugoideae | Ajugeae | *Caryopteris* | *Caryopteris incana* | NC_046409.1 |  |
| Ajugoideae | Ajugeae | *Caryopteris* | *Caryopteris mongholica* | NC_035729.1 |  |
| Ajugoideae | Clerodendreae | *Clerodendrum* | *Clerodendrum bungei* | NC_056141.1 |  |
| Ajugoideae | Clerodendreae | *Clerodendrum* | *Clerodendrum cyrtophyllum* | MW858153.1 |  |
| Ajugoideae | Clerodendreae | *Clerodendrum* | *Clerodendrum mandarinorum* | MN814861.1 |  |
| Ajugoideae | Clerodendreae | *Clerodendrum* | *Clerodendrum chinense* | OM912811 |  |
| Ajugoideae | Clerodendreae | *Clerodendrum* | *Clerodendrum thomsoniae* | OM912812 |  |
| Ajugoideae | Clerodendreae | *Clerodendrum* | *Clerodendrum yunnanense* | MN814862.1 |  |
| Ajugoideae | Clerodendreae | *Clerodendrum* | *Clerodendrum lindleyi* | NC_056199.1 |  |
| Ajugoideae | Clerodendreae | *Clerodendrum* | *Clerodendrum japonicum* | NC_056260.1 |  |
| Ajugoideae | Clerodendreae | *Clerodendrum* | *Clerodendrum trichotomum* | NC_057680.1 |  |
| Nepetoideae | Mentheae | *Dracocephalum* | *Dracocephalum moldavica* | NC_057509.1 |  |
| Nepetoideae | Elsholtzieae | *Elsholtzia* | *Elsholtzia densa* | NC_056994.1 |  |
| Nepetoideae | Ocimeae | *Isodon* | *Isodon serra* | MT317099.1 |  |
| Ajugoideae | Teucrieae | *Teucrium* | *Teucrium ornatum* | MN814864.1 |  |
| Lamioideae | Lamieae | *Lamium* | *Lamium album* | KY562589.1 |  |
| Lamioideae | Lamieae | *Lamium* | *Lamium takeshimense* | MN240520.1 |  |
| Nepetoideae | Ocimeae | *Lavandula* | *Lavandula dentata* | NC_046835.1 |  |
| Outgroup | NA | *NA* | *Mazus pumilus* | NC_042444.1 |  |
| Nepetoideae | Mentheae | *Mentha* | *Mentha canadensis* | NC_044082.1 |  |
| Nepetoideae | Mentheae | *Mentha* | *Mentha longifolia* | NC_032054.1 |  |
| Nepetoideae | Ocimeae | *Ocimum* | *Ocimum gratissimum* | NC_057196.1 |  |
| Outgroup | NA | *NA* | *Phryma leptostachya* | NC_042727.1 |  |
| Lamioideae | Stachydeae | *Phyllostegia* | *Phyllostegia velutina* | NC_029820.1 |  |
| Lamioideae | Pogostemoneae | *Pogostemon* | *Pogostemon stellatus* | NC_031434.1 |  |
| Premnoideae | NA | *Premna* | *Premna microphylla* | NC_026291.1 |  |
| Ajugoideae | Ajugeae | *Pseudocaryopteris* | *Pseudocaryopteris paniculata* | MN814866.1 |  |
| Ajugoideae | Rotheceae | *Rotheca* | *Rotheca myricoides* | NC_059906.1 |  |
| Ajugoideae | Rotheceae | *Rotheca* | *Rotheca serrata* | MT473776.1 |  |
| Nepetoideae | Mentheae | *Salvia* | *Salvia miltiorrhiza* | NC_020431.1 |  |
| Nepetoideae | Mentheae | *Salvia* | *Salvia sclarea* | NC_050900.1 |  |
| Ajugoideae | Teucrieae | *Schnabelia* | *Schnabelia oligophylla* | MT473777.1 |  |
| Ajugoideae | Teucrieae | *Schnabelia* | *Schnabelia tetrodonta* | MW928532.1 |  |
| Scutellarioideae | NA | *Scutellaria* | *Scutellaria amoena* | NC_057255.1 |  |
| Scutellarioideae | NA | *Scutellaria* | *Scutellaria indica* | MN047312.1 |  |
| Stachydeae | NA | *Stachys* | *Stachys byzantina* | NC_029825.1 |  |
| Ajugoideae | Teucrieae | *Teucrium* | *Teucrium mascatense* | NC_044073.1 |  |
| Ajugoideae | Teucrieae | *Teucrium* | *Teucrium omeiense* | MN814871.1 |  |
| Nepetoideae | Mentheae | *Thymus* | *Thymus japonicus* | NC_046822.1 |  |
| Viticoideae | NA | *Vitex* | *Vitex negundo* | NC_057235.1 |  |
