# Supplementary material for: Conserved chloroplast genome sequences of the genus Clerodendrum Linn. (Lamiaceae) as a super-barcode
Source: PLoS One. 2023 Feb 9;18(2):e0277809. doi: 10.1371/journal.pone.0277809 (PMC9910634; doi:10.1371/journal.pone.0277809)
Supplement: S2 Table — (DOCX) [file pone.0277809.s002.docx]

**S2 Table. List of genes in the chloroplast genome of *C. chinense* and *C.*** ***thomsoniae***

| Gene function | Gene classification | Gene names |
| --- | --- | --- |
| rRNA | rRNA genes | *rrn16S*(×2)*, rrn23S*(×2)*, rrn5S*(×2)*, rrn4.5S*(×2) |
| tRNA | tRNA genes | *28trn genes* |
| Self-replication | Large subunit of ribosome | *rpl14, rpl16, rpl2*(×2)*, rpl20, rpl22, rpl23*(×2)*, rpl32, rpl33, rpl36* |
|  | DNA dependent RNA polymerase | *rpoA, rpoB, rpoC1, rpoC2* |
|  | Small subunit of ribosome | *rps11, rps12*(×2)*, rps14, rps15, rps16, rps18, rps19, rps2, rps3, rps4, rps7*(×2)*, rps8* |
| Photosynthesis | Subunits of ATP synthase | *atpA, atpB, atpE, atpF, atpH, atpI* |
|  | Subunits of photosystem II | *psbA, psbB, psbC, psbD, psbE, psbF, psbI, psbJ, psbK, psbL, psbM, psbN, psbT, psbZ, ycf3* |
|  | Subunits of NADH-dehydrogenase | *ndhA, ndhB*(×2)*, ndhC, ndhD, ndhE, ndhF, ndhG, ndhH, ndhI, ndhJ, ndhK* |
|  | Subunits of cytochrome b/f complex | *petA, petB, petD, petG, petL, petN* |
|  | Subunits of photosystem I | *psaA, psaB, psaC, psaI, psaJ* |
|  | Subunit of rubisco | *rbcL* |
| Other functions | Maturase | *matK* |
|  | Protease | *clpP* |
|  | Envelope membrane protein | *cemA* |
|  | Subunit of Acetyl-CoA-carboxylase | *accD* |
|  | c-type cytochrom synthesis gene | *ccsA* |
| Unknown function | | *ycf1*(×2)*, ycf15*(×2)*,ycf2*(×2)*, ycf4* |
