# Supplementary material for: Conserved chloroplast genome sequences of the genus Clerodendrum Linn. (Lamiaceae) as a super-barcode
Source: PLoS One. 2023 Feb 9;18(2):e0277809. doi: 10.1371/journal.pone.0277809 (PMC9910634; doi:10.1371/journal.pone.0277809)
Supplement: S3 Table — (DOCX) [file pone.0277809.s003.docx]

**S3 Table. Intron and exon positions and lengths of chloroplast genes in *C. chinense***

| Gene | Strand | Start | End | Length（bp） | | | | |
| --- | --- | --- | --- | --- | --- | --- | --- | --- |
|  |  |  |  | ExonI | IntronI | ExonII | IntronII | ExonIII |
| *trnK-UUU* | - | 1671 | 4256 | 37 | 2514 | 35 |  |  |
| *rps16* | - | 4920 | 6062 | 40 | 901 | 202 |  |  |
| *trnG-UCC* | + | 9272 | 10036 | 23 | 694 | 48 |  |  |
| *atpF* | - | 12032 | 13260 | 145 | 674 | 410 |  |  |
| *rpoC1* | - | 21087 | 23901 | 430 | 757 | 1628 |  |  |
| *ycf3* | - | 42311 | 44251 | 126 | 714 | 228 | 720 | 153 |
| *trnL-UAA* | + | 47078 | 47646 | 35 | 485 | 49 |  |  |
| *trnV-UAC* | - | 51176 | 51838 | 38 | 590 | 35 |  |  |
| *clpP* | - | 69465 | 71426 | 71 | 738 | 294 | 633 | 226 |
| *petB* | + | 74299 | 75671 | 6 | 725 | 642 |  |  |
| *petD* | + | 75860 | 77095 | 8 | 753 | 475 |  |  |
| *rpl16* | - | 80520 | 81785 | 9 | 858 | 399 |  |  |
| *rpl2* | - | 83454 | 84953 | 396 | 573 | 531 |  |  |
| *ndhB* | - | 93675 | 95886 | 775 | 679 | 758 |  |  |
| *trnI-GAU* | + | 101149 | 102169 | 37 | 949 | 35 |  |  |
| *trnA-UGC* | + | 102234 | 103113 | 38 | 807 | 35 |  |  |
| *ndhA* | - | 118007 | 120013 | 553 | 915 | 539 |  |  |
| *trnA-UGC* | - | 132364 | 133243 | 38 | 807 | 35 |  |  |
| *trnI-GAU* | - | 133308 | 134328 | 37 | 949 | 35 |  |  |
| *ndhB* | + | 139591 | 141802 | 775 | 679 | 758 |  |  |
| *rpl2* | + | 150524 | 152023 | 396 | 573 | 531 |  |  |

Note: "+" indicates the positive stand; "-" indicates the negative strand
