# Supplementary material for: Conserved chloroplast genome sequences of the genus Clerodendrum Linn. (Lamiaceae) as a super-barcode
Source: PLoS One. 2023 Feb 9;18(2):e0277809. doi: 10.1371/journal.pone.0277809 (PMC9910634; doi:10.1371/journal.pone.0277809)
Supplement: S4 Table — (DOCX) [file pone.0277809.s004.docx]

**S4 Table. Intron and exon positions and lengths of chloroplast genes in *C. thomsoniae***

| Gene | Strand | Start | End | Length（bp） | | | | |
| --- | --- | --- | --- | --- | --- | --- | --- | --- |
|  |  |  |  | ExonI | IntronI | ExonII | IntronII | ExonIII |
| *trnK-UUU* | - | 1714 | 4285 | 37 | 2500 | 35 |  |  |
| *rps16* | - | 4935 | 6067 | 40 | 891 | 202 |  |  |
| *trnS-CGA* | + | 8972 | 9736 | 23 | 694 | 48 |  |  |
| *atpF* | - | 11704 | 12945 | 145 | 687 | 410 |  |  |
| *rpoC1* | - | 20731 | 23546 | 430 | 758 | 1628 |  |  |
| *ycf3* | - | 42004 | 43943 | 132 | 707 | 228 | 720 | 153 |
| *trnL-UAA* | + | 46483 | 47052 | 35 | 486 | 49 |  |  |
| *trnV-UAC* | - | 50621 | 51283 | 38 | 590 | 35 |  |  |
| *clpP* | - | 68933 | 70888 | 71 | 729 | 294 | 636 | 226 |
| *petB* | + | 73745 | 75108 | 6 | 716 | 642 |  |  |
| *rpl2* | - | 75297 | 76502 | 8 | 723 | 475 |  |  |
| *rpl2* | - | 79931 | 81183 | 9 | 845 | 399 |  |  |
| *ndhB* | - | 82837 | 84328 | 396 | 565 | 531 |  |  |
| *trnE-UUC* | + | 93032 | 95243 | 775 | 679 | 758 |  |  |
| *trnA-UGC* | + | 100380 | 101399 | 37 | 948 | 35 |  |  |
| *ndhA* | + | 117099 | 119107 | 553 | 917 | 539 |  |  |
| *trnA-UGC* | - | 131453 | 132332 | 38 | 807 | 35 |  |  |
| *trnE-UUC* | - | 132397 | 133416 | 37 | 948 | 35 |  |  |
| *ndhB* | + | 138553 | 140764 | 775 | 679 | 758 |  |  |
| *rpl2* | + | 149468 | 150959 | 396 | 565 | 531 |  |  |

Note: "+" indicates the positive stand; "-" indicates the negative strand
