# Supplementary material for: Conserved chloroplast genome sequences of the genus Clerodendrum Linn. (Lamiaceae) as a super-barcode
Source: PLoS One. 2023 Feb 9;18(2):e0277809. doi: 10.1371/journal.pone.0277809 (PMC9910634; doi:10.1371/journal.pone.0277809)
Supplement: S6 Table — (DOCX) [file pone.0277809.s006.docx]

**S6 Table.** **Distribution of nucleotide SSR sequences in the chloroplast genome of *C. chinense***

| **SSR nr.** | **SSR type** | **SSR sequence** | **Size** | **Start** | **End** | **Location** |
| --- | --- | --- | --- | --- | --- | --- |
| 1 | c | (C)10......(TA)6 | 96 | 5148 | 5243 | Intron (*rps16*) |
| 2 | c | (T)11......(AT)9 | 44 | 6633 | 6676 | *IGS* (((*rps16- trnQ-UUG*)) |
| 3 | p1 | (A)11 | 11 | 6860 | 6870 | *IGS* (((*rps16- trnQ-UUG*)) |
| 4 | p1 | (T)10 | 10 | 8916 | 8925 | *IGS* (((*psbI- trnS-GCU*)) |
| 5 | p1 | (T)10 | 10 | 9068 | 9077 | *IGS* (((*trnS-GCU- trnG-UCC*)) |
| 6 | p1 | (T)10 | 10 | 10043 | 10052 | *IGS* (((*trnG-UCC- trnR-UCU*)) |
| 7 | p1 | (T)15 | 15 | 11954 | 11968 | *IGS* (((*atpA- atpF*)) |
| 8 | c | (C)13(T)11c(A)11 | 36 | 16550 | 16585 | *Intron* (((*rpoC2*)) |
| 9 | p1 | (T)11 | 11 | 18775 | 18785 | *IGS* (((*rpoB- trnC-GCA*)) |
| 10 | p1 | (T)10 | 10 | 27581 | 27590 | *IGS* (((*psbC- trnS-UGA*)) |
| 11 | p1 | (T)10 | 10 | 35251 | 35260 | *IGS* (((*psaA- trnS-GGA*)) |
| 12 | p1 | (T)10 | 10 | 43142 | 43151 | *IGS* (((*psaA- trnS-GGA*)) |
| 13 | p1 | (A)10 | 10 | 44104 | 44113 | *IGS* (((*psaA- trnS-GGA*)) |
| 14 | p1 | (T)11 | 11 | 44396 | 44406 | *IGS* (((*rps4- trnT-UGU*)) |
| 15 | p2 | (TA)7 | 14 | 46125 | 46138 | *IGS* ((*trnT-UGU- trnL-UAA*)) |
| 16 | p1 | (T)15 | 15 | 46623 | 46637 | *IGS* ((*trnT-UGU- trnL-UAA*)) |
| 17 | p1 | (A)13 | 13 | 47061 | 47073 | *Intron* ((*trnL-UAA*)) |
| 18 | p1 | (A)11 | 11 | 47242 | 47252 | *IGS* ((*ndhC- trnV-UAC*)) |
| 19 | p1 | (T)10 | 10 | 50762 | 50771 | *IGS* ((*atpB-rbcL*)) |
| 20 | c | (T)10......(ATA)5 | 69 | 54147 | 54215 | *IGS* ((*rbcL-accD*)) |
| 21 | p1 | (T)10 | 10 | 56858 | 56867 | *IGS* ((*psaI- ycf4*)) |
| 22 | p1 | (T)10 | 10 | 59356 | 59365 | *IGS* ((*rpl20-rps12*)) |
| 23 | p1 | (A)10 | 10 | 68643 | 68652 | *Intron* ((*clpP*)) |
| 24 | p1 | (A)13 | 13 | 70689 | 70701 | *Intron* ((*petB*)) |
| 25 | p1 | (A)10 | 10 | 74507 | 74516 | *Intron* ((*petB*)) |
| 26 | c | (T)12......(G)11 | 76 | 74940 | 75015 | *IGS* ((*rpoA- rps11*)) |
| 27 | p1 | (T)10 | 10 | 78312 | 78321 | *IGS* ((*infA-rps8*)) |
| 28 | p1 | (T)11 | 11 | 79415 | 79425 | *Intron* ((*rpl16*)) |
| 29 | p1 | (A)11 | 11 | 81110 | 81120 | *Intron* ((*trnI-GAU*)) |
| 30 | p1 | (T)10 | 10 | 101623 | 101632 | *IGS* ((*rpl32- trnL-UAG*)) |
| 31 | p1 | (T)10 | 10 | 111441 | 111450 | *IGS* ((*rpl32- trnL-UAG*)) |
| 32 | c | (T)12(G)10 | 22 | 112230 | 112251 | *Intron* ((*ccsA*)) |
| 33 | p1 | (A)11 | 11 | 113279 | 113289 | *CDS* ((*ndhA*)) |
| 34 | p1 | (T)10 | 10 | 118853 | 118862 | *Intron* ((*ycf1*)) |
| 35 | p1 | (T)12 | 12 | 123924 | 123935 | *Intron* ((*ycf1*)) |
| 36 | p1 | (T)11 | 11 | 124324 | 124334 | *Intron* ((*trnI-GAU*)) |
| 37 | p1 | (A)10 | 10 | 133845 | 133854 | *Intron* ((*rrn16S*)) |

Note: IGS, intergenic spacers. Intron, intron of gene. CDS, coding sequences. p1: base number in repeat unit is 1. p2: base number in repeat unit is 2. c: complex repeat unit
