# Supplementary material for: Conserved chloroplast genome sequences of the genus Clerodendrum Linn. (Lamiaceae) as a super-barcode
Source: PLoS One. 2023 Feb 9;18(2):e0277809. doi: 10.1371/journal.pone.0277809 (PMC9910634; doi:10.1371/journal.pone.0277809)
Supplement: S7 Table — (DOCX) [file pone.0277809.s007.docx]

**S7 Table. Statistics on the number of microsatellite repeat sequences in the chloroplast genome of *C. thomsoniae***

| Type of repeat unit | Number of repeating sequences |
| --- | --- |
| A/T | 40 |
| AT/AT | 1 |
| AAT/ATT | 1 |
