# Supplementary material for: Conserved chloroplast genome sequences of the genus Clerodendrum Linn. (Lamiaceae) as a super-barcode
Source: PLoS One. 2023 Feb 9;18(2):e0277809. doi: 10.1371/journal.pone.0277809 (PMC9910634; doi:10.1371/journal.pone.0277809)
Supplement: S8 Table — (DOCX) [file pone.0277809.s008.docx]

**S8 Table. Distribution of nucleotide SSR sequences in the chloroplast genome of *C. thomsoniae*.**

| **SSR nr.** | **SSR type** | **SSR sequence** | **Size** | **Start** | **End** | **Location** |
| --- | --- | --- | --- | --- | --- | --- |
| 1 | p1 | (A)10 | 10 | 3865 | 3874 | *IGS* (*matK-rps16*) |
| 2 | p2 | (TA)6 | 12 | 5238 | 5249 | *Intron^b^* (*rps16*) |
| 3 | p1 | (A)10 | 10 | 6494 | 6503 | *IGS* (*rps16-trnQ-UUG*) |
| 4 | p1 | (T)10 | 10 | 6955 | 6964 | *IGS* (*rps16-trnQ-UUG*) |
| 5 | p1 | (T)10 | 10 | 8811 | 8820 | *IGS* (*trnS-GCU- trnG-UCC*) |
| 6 | p1 | (T)11 | 11 | 9743 | 9753 | *IGS* (*trnG-UCC- trnR-UCU*) |
| 7 | p1 | (T)10 | 10 | 11633 | 11642 | *IGS* (*atpA - atpF*) |
| 8 | p1 | (T)10 | 10 | 12372 | 12381 | *Intron* (*atpF*) |
| 9 | p1 | (T)11 | 11 | 13024 | 13034 | *Intron* (*atpH*) |
| 10 | p1 | (T)11 | 11 | 16228 | 16238 | *Intron* (*rpoC2*) |
| 11 | p1 | (T)11 | 11 | 18419 | 18429 | *Intron* (*rpoC2*) |
| 12 | p1 | (A)10 | 10 | 22885 | 22894 | *Intron* (*rpoC1*) |
| 13 | p1 | (T)10 | 10 | 27219 | 27228 | *IGS* (*rpoB- trnC-GCA*) |
| 14 | p1 | (T)10 | 10 | 31347 | 31356 | *IGS* (*trnT-GGU- psbD*) |
| 15 | p1 | (T)10 | 10 | 31998 | 32007 | *IGS* (*trnT-GGU- psbD*) |
| 16 | p1 | (T)12 | 12 | 34991 | 35002 | *IGS* (*psbC-trnS-UGA*) |
| 17 | p1 | (T)11 | 11 | 42834 | 42844 | *IGS* (*psaA-trnS-GGA*) |
| 18 | c | (A)11......(T)13 | 53 | 44048 | 44100 | *IGS* (*psaA-trnS-GGA*) |
| 19 | p1 | (T)14 | 14 | 45395 | 45408 | *IGS* (*rps4- trnT-UGU*) |
| 20 | p1 | (A)10 | 10 | 46358 | 46367 | *IGS* (*trnT-UGU - trnL-UAA*) |
| 21 | c | (T)10......(ATA)5 | 69 | 53607 | 53675 | *IGS* (*atpB - rbcL*) |
| 22 | p1 | (T)11 | 11 | 56322 | 56332 | *Intron* (*accD*) |
| 23 | p1 | (T)12 | 12 | 58822 | 58833 | *IGS* (*psaI- ycf4*) |
| 24 | p1 | (A)11 | 11 | 62971 | 62981 | *IGS* (*petA- psbJ*) |
| 25 | p1 | (T)10 | 10 | 69435 | 69444 | *Intron* (*clpP*) |
| 26 | p1 | (A)11 | 11 | 70160 | 70170 | *Intron* (*clpP*) |
| 27 | p1 | (A)10 | 10 | 73947 | 73956 | *Intron* (*petB*) |
| 28 | p1 | (T)10 | 10 | 78812 | 78821 | *IGS* (*infA-rps8*) |
| 29 | p1 | (T)15 | 15 | 79324 | 79338 | *IGS* (*rps8- rpl14*) |
| 30 | p1 | (A)11 | 11 | 80499 | 80509 | *Intron* (*rpl16*) |
| 31 | p1 | (A)10 | 10 | 95266 | 95275 | *IGS* (*ndhB- rps7*) |
| 32 | p1 | (T)10 | 10 | 108278 | 108287 | *IGS* (*ycf1- ndhF*) |
| 33 | p1 | (T)11 | 11 | 110828 | 110838 | *IGS* (*ndhF- rpl32*) |
| 34 | p1 | (A)11 | 11 | 111395 | 111405 | *IGS* (*rpl32- trnL-UAG*) |
| 35 | p1 | (A)10 | 10 | 111745 | 111754 | *IGS* (*trnL-UAG- ccsA*) |
| 36 | p1 | (A)11 | 11 | 112419 | 112429 | *CDS* (*ccsA*) |
| 37 | p1 | (A)10 | 10 | 117934 | 117943 | *CDS* (*ndhA*) |
| 38 | p1 | (T)10 | 10 | 118228 | 118237 | *CDS* (*ndhA*) |
| 39 | p1 | (T)12 | 12 | 123001 | 123012 | *CDS* (*ycf1*) |
| 40 | p1 | (T)10 | 10 | 138521 | 138530 | *IGS* (*rps7- ndhB*) |

Note: IGS, intergenic spacers. Intron, intron of gene. CDS, coding sequences. p1: base number in repeat unit is 1. p2: base number in repeat unit is 2. c: complex repeat unit
