# Supplementary material for: Conserved chloroplast genome sequences of the genus Clerodendrum Linn. (Lamiaceae) as a super-barcode
Source: PLoS One. 2023 Feb 9;18(2):e0277809. doi: 10.1371/journal.pone.0277809 (PMC9910634; doi:10.1371/journal.pone.0277809)
Supplement: S9 Table — (DOCX) [file pone.0277809.s009.docx]

**S9 Table. Tandem repeat sequence statistics of the chloroplast genome of *C. chinense***

| **Start-End** | **Location** | **Length**  **of Repeat**  **（bp）** | **Copy Number**  **of Repeat** | **Corresponding**  **Sequence**  **（bp）** | **Matching score of repeat（%）** | **Percentage of lost units（%）** | **Number of bp** | | | |
| --- | --- | --- | --- | --- | --- | --- | --- | --- | --- | --- |
|  |  |  |  |  |  |  | **A** | **C** | **G** | **T** |
| 6280-6321 | *IGS* (*rps16-trnQ-UUG*) | 18 | 2.4 | 18 | 88 | 4 | 61 | 4 | 19 | 14 |
| 15811-15836 | *IGS* (*trnD-GUC-trnY-GUA*) | 13 | 2 | 13 | 100 | 0 | 30 | 7 | 0 | 61 |
| 30556-30599 | *IGS* (*rps4 - trnT-UGU*) | 22 | 2 | 22 | 100 | 0 | 54 | 13 | 18 | 13 |
| 46046-46071 | *IGS* (*ycf4 - cemA*) | 13 | 2 | 13 | 100 | 0 | 30 | 7 | 23 | 38 |
| 60483-60507 | *CDS* (*rps18*) | 12 | 2.1 | 12 | 100 | 0 | 52 | 32 | 0 | 16 |
| 67479-67519 | *Intron* (*ycf2*) | 21 | 2 | 21 | 90 | 0 | 29 | 24 | 9 | 36 |
| 88157-88211 | *Intron* (*ycf2*) | 24 | 2.5 | 22 | 85 | 8 | 14 | 23 | 12 | 49 |
| 88179-88246 | *Intron* (*ycf2*) | 21 | 3.2 | 21 | 77 | 8 | 11 | 22 | 10 | 55 |
| 90572-90667 | *IGS* (*rps7- trnV-GAC*) | 18 | 5.3 | 18 | 100 | 0 | 28 | 11 | 27 | 33 |
| 98378-98412 | *IGS* (*rrn4.5S- rrn5S*) | 17 | 2 | 18 | 94 | 5 | 28 | 0 | 22 | 48 |
| 106337-106397 | *IGS* (*rpl32- trnL-UAG*) | 31 | 2 | 31 | 96 | 0 | 39 | 22 | 9 | 27 |
| 112663-112687 | *IGS* (*rps15- ycf1*) | 11 | 2.3 | 11 | 100 | 0 | 24 | 0 | 8 | 68 |
| 121725-121750 | *Intron* (*ycf1*) | 13 | 2 | 13 | 100 | 0 | 53 | 0 | 23 | 23 |
| 125128-125171 | *IGS* (*rrn5S- rrn4.5S*) | 14 | 3.2 | 14 | 80 | 3 | 0 | 13 | 9 | 77 |
| 129080-129140 | *IGS* (*trnV-GAC - rps7*) | 31 | 2 | 31 | 96 | 0 | 27 | 9 | 22 | 39 |
| 137065-137099 | *Intron* (*ycf2*) | 17 | 2 | 18 | 94 | 5 | 48 | 22 | 0 | 28 |
| 144810-144905 | *Intron* (*ycf2*) | 18 | 5.3 | 18 | 100 | 0 | 33 | 27 | 11 | 28 |
| 147249-147302 | *Intron* (*ycf2*) | 21 | 2.6 | 21 | 82 | 11 | 59 | 11 | 20 | 9 |
| 147231-147320 | *Intron* (*ycf2*) | 21 | 4.1 | 22 | 75 | 8 | 53 | 11 | 23 | 12 |
