# Supplementary material for: Conserved chloroplast genome sequences of the genus Clerodendrum Linn. (Lamiaceae) as a super-barcode
Source: PLoS One. 2023 Feb 9;18(2):e0277809. doi: 10.1371/journal.pone.0277809 (PMC9910634; doi:10.1371/journal.pone.0277809)
Supplement: S10 Table — (DOCX) [file pone.0277809.s010.docx]

**S10 Table. Tandem repeat sequence statistics of the chloroplast genome of *C. thomsoniae***

| **Start-End** | **Location** | **Length**  **of Repeat**  **（bp）** | **Copy Number**  **of Repeat** | **Corresponding**  **Sequence**  **（bp）** | **Matching score of repeat（%）** | **Percentage of lost units（%）** | **Number of bp** | | | |
| --- | --- | --- | --- | --- | --- | --- | --- | --- | --- | --- |
|  |  |  |  |  |  |  | **A** | **C** | **G** | **T** |
| 6274-6315 | *IGS* (*rps16-trnQ-UUG*) | 18 | 2.4 | 18 | 88 | 4 | 61 | 4 | 19 | 14 |
| 32006-32032 | *IGS* (*trnT-GGU- psbD*) | 14 | 1.9 | 14 | 100 | 0 | 37 | 3 | 7 | 51 |
| 45480-45505 | *IGS* (*rps4- trnT-UGU*) | 13 | 2 | 13 | 100 | 0 | 23 | 7 | 30 | 38 |
| 50097-50136 | *IGS* (*ndhC- trnV-UAC*) | 21 | 1.9 | 21 | 100 | 0 | 35 | 2 | 0 | 62 |
| 51659-51698 | *IGS* (*trnM-CAU- atpE*) | 15 | 2.7 | 15 | 88 | 11 | 12 | 12 | 27 | 47 |
| 66407-66431 | *IGS* (*psaJ- rpl33*) | 12 | 2.1 | 12 | 100 | 0 | 32 | 8 | 0 | 60 |
| 87538-87592 | *CDS* (*ycf2*) | 24 | 2.5 | 22 | 85 | 8 | 14 | 23 | 12 | 49 |
| 87560-87627 | *CDS* (*ycf2*) | 21 | 3.2 | 21 | 77 | 8 | 11 | 22 | 10 | 55 |
| 89953-90030 | *CDS* (*ycf2*) | 18 | 4.3 | 18 | 100 | 0 | 28 | 11 | 26 | 33 |
| 105573-105633 | *IGS* (*rrn4.5S- rrn5S*) | 31 | 2 | 31 | 93 | 0 | 39 | 24 | 9 | 26 |
| 124205-124248 | *CDS* (*ycf1*) | 14 | 3.2 | 14 | 80 | 3 | 0 | 13 | 9 | 77 |
| 128163-128223 | *IGS* (*rrn5S- rrn4.5S*) | 31 | 2 | 31 | 93 | 0 | 26 | 9 | 24 | 39 |
| 136155-136200 | *IGS* (*trnV-GAC- rps7*) | 23 | 2 | 23 | 86 | 0 | 60 | 6 | 6 | 26 |
| 143766-143843 | *CDS* (*ycf2*) | 18 | 4.3 | 18 | 100 | 0 | 33 | 26 | 11 | 28 |
| 146187-146240 | *CDS* (*ycf2*) | 21 | 2.6 | 21 | 82 | 11 | 59 | 11 | 20 | 9 |
| 146169-146258 | *CDS* (*ycf2*) | 21 | 4.1 | 22 | 75 | 8 | 53 | 11 | 23 | 12 |
