# Supplementary material for: Conserved chloroplast genome sequences of the genus Clerodendrum Linn. (Lamiaceae) as a super-barcode
Source: PLoS One. 2023 Feb 9;18(2):e0277809. doi: 10.1371/journal.pone.0277809 (PMC9910634; doi:10.1371/journal.pone.0277809)
Supplement: S11 Table — (DOCX) [file pone.0277809.s011.docx]

**S11 Table. Characteristic values of scattered repetitive sequences in the chloroplast genome of *C. chinense***

| **Length of**  **Repeat Unit Ⅰ**  **/bp** | **Repeat Unit Ⅰ Start** | **Length of Repeat Unit Ⅱ/bp** | **Repeat Unit Ⅱ**  **Start** | **Repeat type** | **Gap of repeat unit** | ***e*-value** |
| --- | --- | --- | --- | --- | --- | --- |
| 82 | 87358 | 82 | 87376 | D | -1 | 6.56E-38 |
| 82 | 87358 | 82 | 141592 | P | -1 | 6.56E-38 |
| 82 | 87376 | 82 | 141610 | P | -1 | 6.56E-38 |
| 82 | 141592 | 82 | 141610 | D | -1 | 6.56E-38 |
| 64 | 87358 | 64 | 87394 | D | -1 | 3.52E-27 |
| 64 | 87358 | 64 | 141592 | P | -1 | 3.52E-27 |
| 64 | 87394 | 64 | 141628 | P | -1 | 3.52E-27 |
| 64 | 141592 | 64 | 141628 | D | -1 | 3.52E-27 |
| 56 | 26438 | 56 | 26438 | P | -2 | 1.66E-20 |
| 48 | 70459 | 48 | 70459 | P | 0 | 7.87E-20 |
| 46 | 87358 | 46 | 87412 | D | -1 | 1.74E-16 |
| 46 | 87358 | 46 | 141592 | P | -1 | 1.74E-16 |
| 46 | 87412 | 46 | 141646 | P | -1 | 1.74E-16 |
| 46 | 141592 | 46 | 141646 | D | -1 | 1.74E-16 |
| 41 | 94302 | 41 | 115370 | D | 0 | 1.29E-15 |
| 41 | 115370 | 41 | 134707 | P | 0 | 1.29E-15 |
| 44 | 110636 | 44 | 110636 | P | -2 | 1.72E-13 |
| 39 | 40236 | 39 | 94304 | D | -1 | 2.41E-12 |
| 39 | 40236 | 39 | 115372 | D | -1 | 2.41E-12 |
| 39 | 40236 | 39 | 134707 | P | -1 | 2.41E-12 |
| 42 | 59860 | 42 | 59860 | P | -2 | 2.50E-12 |
| 39 | 38763 | 39 | 38763 | P | -3 | 5.09E-09 |
| 30 | 8531 | 30 | 41769 | P | 0 | 5.41E-09 |
| 37 | 4699 | 37 | 4699 | P | -3 | 6.92E-08 |
| 30 | 103123 | 30 | 103154 | D | -1 | 4.87E-07 |
| 30 | 103123 | 30 | 125866 | P | -1 | 4.87E-07 |
| 30 | 103154 | 30 | 125897 | P | -1 | 4.87E-07 |
| 30 | 125866 | 30 | 125897 | D | -1 | 4.87E-07 |
| 34 | 35256 | 34 | 37480 | D | -3 | 3.41E-06 |
| 31 | 110487 | 31 | 110535 | P | -2 | 5.66E-06 |
| 32 | 8529 | 32 | 32178 | D | -3 | 4.53E-05 |
| 32 | 27332 | 32 | 27354 | D | -3 | 4.53E-05 |
| 31 | 87355 | 31 | 87427 | D | -3 | 1.64E-04 |
| 31 | 87355 | 31 | 141592 | P | -3 | 1.64E-04 |
| 31 | 87427 | 31 | 141664 | P | -3 | 1.64E-04 |
| 31 | 141589 | 31 | 141661 | D | -3 | 1.64E-04 |
| 30 | 5147 | 30 | 71770 | P | -3 | 5.93E-04 |
| 30 | 10004 | 30 | 33044 | D | -3 | 5.93E-04 |
| 30 | 32180 | 30 | 41769 | P | -3 | 5.93E-04 |
| 30 | 51231 | 30 | 62032 | P | -3 | 5.93E-04 |
| 30 | 84961 | 30 | 85003 | D | -3 | 5.93E-04 |
| 30 | 84961 | 30 | 144017 | P | -3 | 5.93E-04 |
| 30 | 85003 | 30 | 144059 | P | -3 | 5.93E-04 |
| 30 | 144017 | 30 | 144059 | D | -3 | 5.93E-04 |

Note: P indicates palindromic repeat; D indicates direct repeat.
