# Supplementary material for: Conserved chloroplast genome sequences of the genus Clerodendrum Linn. (Lamiaceae) as a super-barcode
Source: PLoS One. 2023 Feb 9;18(2):e0277809. doi: 10.1371/journal.pone.0277809 (PMC9910634; doi:10.1371/journal.pone.0277809)
Supplement: S12 Table — (DOCX) [file pone.0277809.s012.docx]

**S12 Table. Characteristic values of scattered repetitive sequences in the chloroplast genome of *C. thomsoniae.***

| **Length of**  **Repeat Unit Ⅰ**  **/bp** | **Repeat Unit Ⅰ Start** | **Length of Repeat Unit Ⅱ/bp** | **Repeat Unit Ⅱ**  **Start** | **Repeat type** | **Gap of repeat unit** | ***e*-value** |
| --- | --- | --- | --- | --- | --- | --- |
| 64 | 89952 | 64 | 89970 | D | -1 | 3.62E-27 |
| 64 | 89952 | 64 | 143761 | P | -1 | 3.62E-27 |
| 64 | 89970 | 64 | 143779 | P | -1 | 3.62E-27 |
| 64 | 143761 | 64 | 143779 | D | -1 | 3.62E-27 |
| 46 | 89952 | 46 | 89988 | D | -1 | 1.79E-16 |
| 46 | 89952 | 46 | 143761 | P | -1 | 1.79E-16 |
| 46 | 89988 | 46 | 143797 | P | -1 | 1.79E-16 |
| 46 | 143761 | 46 | 143797 | D | -1 | 1.79E-16 |
| 42 | 62532 | 42 | 62532 | P | 0 | 3.32E-16 |
| 48 | 73118 | 48 | 73118 | P | -2 | 8.22E-16 |
| 41 | 96871 | 41 | 117675 | D | 0 | 1.33E-15 |
| 41 | 117675 | 41 | 136883 | P | 0 | 1.33E-15 |
| 44 | 112951 | 44 | 112951 | P | -2 | 1.77E-13 |
| 39 | 41696 | 39 | 41696 | P | -1 | 2.48E-12 |
| 39 | 43142 | 39 | 96873 | D | -1 | 2.48E-12 |
| 39 | 43142 | 39 | 117677 | D | -1 | 2.48E-12 |
| 39 | 43142 | 39 | 136883 | P | -1 | 2.48E-12 |
| 41 | 75502 | 41 | 75502 | P | -3 | 3.82E-10 |
| 30 | 8249 | 30 | 44422 | P | 0 | 5.57E-09 |
| 37 | 4725 | 37 | 4725 | P | -3 | 7.13E-08 |
| 32 | 59108 | 32 | 59108 | P | -2 | 1.55E-06 |
| 34 | 38189 | 34 | 40413 | D | -3 | 3.51E-06 |
| 30 | 105572 | 30 | 105603 | D | -2 | 2.18E-05 |
| 30 | 105572 | 30 | 128162 | P | -2 | 2.18E-05 |
| 30 | 105603 | 30 | 128193 | P | -2 | 2.18E-05 |
| 30 | 128162 | 30 | 128193 | D | -2 | 2.18E-05 |
| 32 | 8247 | 32 | 35132 | D | -3 | 4.66E-05 |
| 31 | 29453 | 31 | 29478 | P | -3 | 1.69E-04 |
| 31 | 89949 | 31 | 90003 | D | -3 | 1.69E-04 |
| 31 | 89949 | 31 | 143761 | P | -3 | 1.69E-04 |
| 31 | 90003 | 31 | 143815 | P | -3 | 1.69E-04 |
| 31 | 143758 | 31 | 143812 | D | -3 | 1.69E-04 |
| 30 | 35134 | 30 | 44422 | P | -3 | 6.10E-04 |
| 30 | 87555 | 30 | 87597 | D | -3 | 6.10E-04 |
| 30 | 87555 | 30 | 146168 | P | -3 | 6.10E-04 |
| 30 | 87597 | 30 | 146210 | P | -3 | 6.10E-04 |
| 30 | 146168 | 30 | 146210 | D | -3 | 6.10E-04 |

Note: P indicates palindromic repeat; D indicates direct repeat.
