# Supplementary material for: Conserved chloroplast genome sequences of the genus Clerodendrum Linn. (Lamiaceae) as a super-barcode
Source: PLoS One. 2023 Feb 9;18(2):e0277809. doi: 10.1371/journal.pone.0277809 (PMC9910634; doi:10.1371/journal.pone.0277809)
Supplement: S1 Fig — (DOCX) [file pone.0277809.s013.docx]

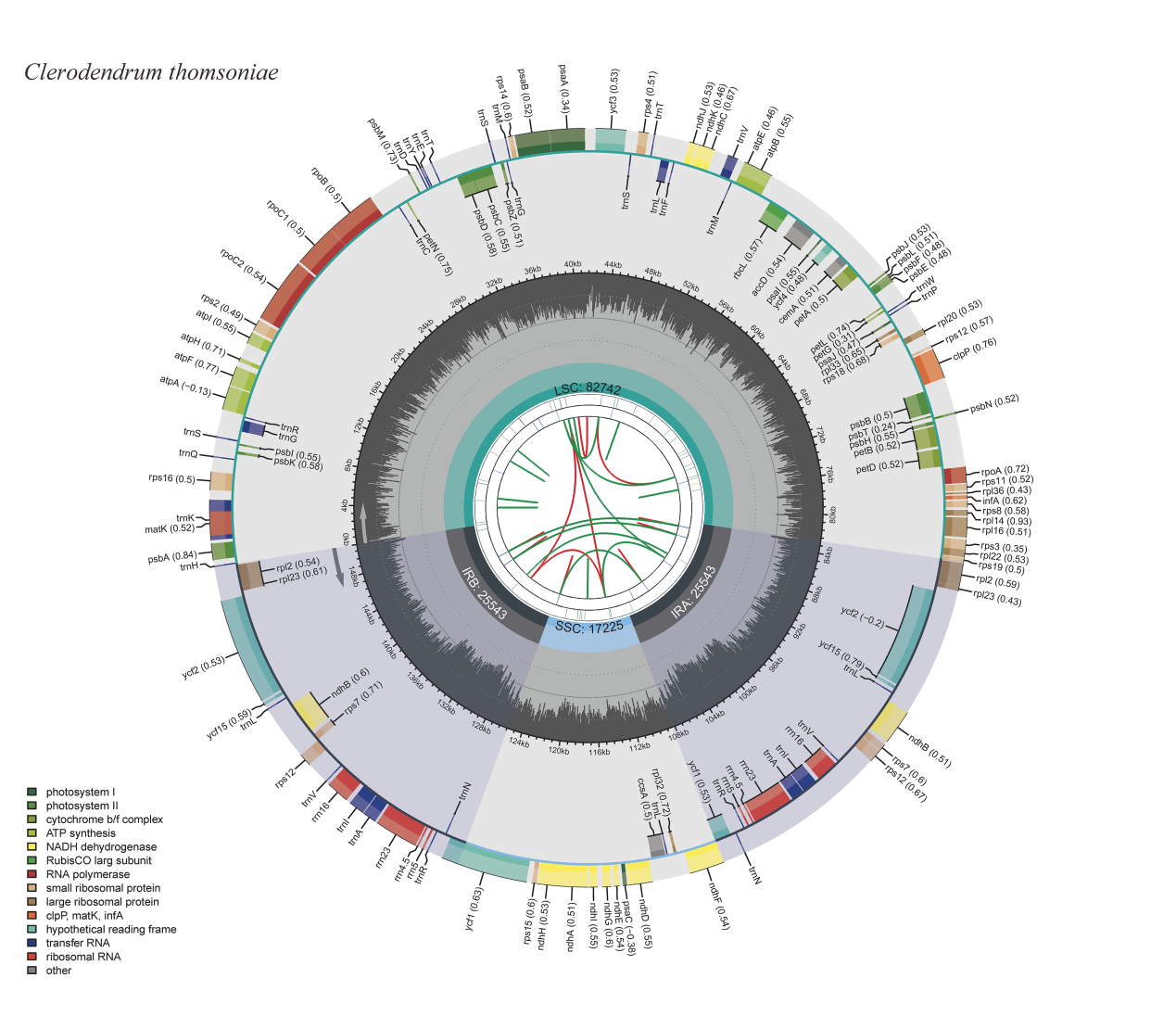


**S1 Fig. Map of the chloroplast genome of *C. thomsoniae* using CPGview-RSG.**

There are six rings on the diagram from the center outwards. The first ring is used to indicate the position of forward (red arcs) and reverse (green arcs) repeats. The second ring is used to indicate tandem repeats (short columns). The third ring is used to indicate microsatellite repeats (short columns). The fourth shaded ring indicates each part and the length of the single short copy (SSC), inverted repeat (IRa and IRb), and large single-copy (LSC) regions. The optional shaded area stretching from the inner sphere toward the outer circle marks the IR regions The fifth ring indicates the GC content of the chloroplast genome. The outer ring shows the gene names and their optional codon usage bias. The genes are colored based on their functional categories.
